# Rolling out RapidPlan: What we've learnt

**DOI:** 10.1002/jmrs.420

**Published:** 2020-09-03

**Authors:** Kirsten van Gysen, James O'Toole, Andrew Le, Kenny Wu, Thilo Schuler, Brian Porter, John Kipritidis, John Atyeo, Chris Brown, Thomas Eade

**Affiliations:** ^1^ Northern Sydney Cancer Centre Royal North Shore Hospital St Leonards NSW Australia; ^2^ NHMRC Clinical Trial Centre University of Sydney Camperdown NSW Australia; ^3^ Northern Clinical School University of Sydney Camperdown NSW Australia

**Keywords:** Knowledge‐based planning, radiation therapy, RapidPlan

## Abstract

**Introduction:**

RapidPlan (RP), a knowledge‐based planning system, aims to consistently improve plan quality and efficiency in radiotherapy. During the early stages of implementation, some of the challenges include knowing how to optimally train a model and how to integrate RP into a department. We discuss our experience with the implementation of RP into our institution.

**Methods:**

We reviewed all patients planned using RP over a 7‐month period following inception in our department. Our primary outcome was clinically acceptable plans (used for treatment) with secondary outcomes including model performance and a comparison of efficiency and plan quality between RP and manual planning (MP).

**Results:**

Between November 2017 and May 2018, 496 patients were simulated, of which 217 (43.8%) had an available model. RP successfully created a clinically acceptable plan in 87.2% of eligible patients. The individual success of the 24 models ranged from 50% to 100%, with more than 90% success in 15 (62.5%) of the models. In 40% of plans, success was achieved on the 1st optimisation. The overall planning time with RP was reduced by up to 95% compared with MP times. The quality of the RP plans was at least equivalent to historical MP plans in terms of target coverage and organ at risk constraints.

**Conclusion:**

While initially time‐consuming and resource‐intensive to implement, plans optimised with RP demonstrate clinically acceptable plan quality, while significantly improving the efficiency of a department, suggesting RP and its application is a highly effective tool in clinical practice.

## Graphical abstract

Knowledge‐based planning, RapidPlan (RP), aims to consistently improve plan quality and efficiency in a radiotherapy department. We discuss our experience with the implementation, validation and outcomes associated with the use of RP in a single institution. While initially time‐consuming and resource‐intensive to implement, plans optimised with RP demonstrate clinically acceptable plan quality, while significantly improving the efficiency of a department, suggesting RP and its application is a highly effective tool in clinical practice.

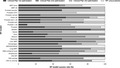

## Introduction

With increasing complexity of radiotherapy, it can be a challenge to consistently produce high‐quality radiation plans which accurately deliver dose to the target while sparing organs at risk (OAR).^1^ Inverse planning with intensity‐modulated radiation therapy (IMRT) and volumetric modulated arc therapy (VMAT) has improved target coverage and normal tissue sparing,^1^, ^2^ but the optimisation process can be time‐consuming. On completion of a treatment plan, if dose constraints fulfil department protocol requirements, a plan is approved, but there may still be potential for improvement.

RapidPlan (RP) (Varian Medical Systems, Palo, Alto, CA) is a knowledge‐based planning (KBP) tool which aims to achieve plan consistency, improved plan quality and greater efficiency in a radiotherapy department.^3^ It also has a role in plan quality analysis. For each treatment subsite, a RP model is trained using a library of high‐quality previously treated plans. At the time of planning, the model provides estimated dose volume histograms (DVH) for OARs with optimisation objectives that can be used as a starting point and a guide for subsequent plans, therefore streamlining the planning process.

A number of publications have demonstrated the effectiveness of RP when compared to manual planning for a variety of anatomical subsites.^4^, ^5^, ^6^, ^7^ High‐quality RP models result in improved plan quality, more optimal target coverage, reduced OAR doses and substantially reduced planning times.^4^, ^5^, ^6^, ^7^ Despite this, due to the time involved in creating RP models and the lack of available resources, many departments have not started using RP routinely. The practical implications around the broad implementation of RP in a clinical environment have yet to be reported. The aim of this study was to report on our experience with the early implementation of RP in our department and the practical implications associated with it. The outcomes are not the results of a direct comparison and therefore there are limitations to our report, but it should be used as a yardstick to guide further research in the area and to inform departments of potential outcomes that can be achieved when using RP.

## Materials and Methods

The data for this study were obtained from our ethically approved research database (NSLHD reference: RESP/15/255). All patients in this study had consented to be included in the database. Varian RapidPlan version 13.6 was implemented in the department and over a period of 12 months 24 models were created. To evaluate the performance of RP and the practical impact that this tool had on our department, we reviewed all plans created with RP since its inception in our department. The primary outcome was clinically acceptable plans, defined as a plan approved for treatment by a Radiation Oncologist (RO) according to recognised treatment protocols.^8^ Secondary outcomes included model performance and a comparison of efficiency when planning manually and with RP. To ensure that the plans created with RP were not inferior in quality to previously accepted department treatment plans, we performed a simple dosimetric planning comparison between RP and manual plans (MP). All RP models used in the study had been validated according to department policy and approved for clinical use. The validation process involved a cohort of 10 patients (not used for training the model) who were replanned using the RP optimisation objectives and a single pass through the optimiser with no intervention from the planner. The RP plan was evaluated against the treated plan by ROs. The model was deemed acceptable if the target volumes had the same or better coverage and OARs had the equivalent or less dose than the treated plan in 90% of the plans.^9^, ^10^

### Clinically acceptable plans

The percentage of clinically acceptable plans produced by a model was used as an indicator of its success. Plan evaluation was carried out using published national guidelines.^8^ A plan was considered to be clinically acceptable if it met published target coverage and OAR objectives. In certain circumstances where acceptable coverage of all structures could not be simultaneously achieved, a plan could be approved at the discretion of the treating radiation oncologist, by sacrificing some PTV coverage in favour of OAR constraints or by accepting slightly higher OAR doses if considered to be safe.

### Model performance

The performance of each RP model was assessed by the number of optimisations that were required to create a clinically acceptable plan, with the ideal being a single optimisation. During planning, RP was allowed up to three optimisation attempts. If a plan did not meet plan objectives following the first optimisation, optimisation objectives were adjusted to suit the clinical protocol and a subsequent optimisation was performed. Plans that were unsuccessful after three optimisations with RP were defined as a failure of RP and the plan was completed manually, usually by starting the planning process from the beginning.

### RP and MP efficiency comparison

The efficiency of RP and MP was compared by assessing the difference in the number of optimisations needed and the overall planning time required to achieve a clinically acceptable plan for the different subsites. Optimisations for both MP and RP were done on the same software and servers. Planning time was defined as the total time taken to achieve a clinically acceptable treatment plan. It was measured from the start of the first optimisation to the end of the calculation time of the clinically acceptable plan. The MP data for planning times and the number of optimisations were collected in our department during routine manual planning as part of a quality analysis timing study at the time of RP implementation. The RP data for planning time and number of optimisations were collected as plans were created using RP. There were 10 treatment sites which had both MP data and a corresponding RP model for a comparison to be made.

### RP and MP Plan Quality comparison

To ensure that the plans created with RP were not inferior in quality to those manually planned, we performed a dosimetric planning comparison. Clinically acceptable plans that had been created using RP models were compared to historical manually created plans from the same subsite that had been used for treatment within the previous 3 years. MPs from within this time frame were chosen as there had been no change in objectives for plan acceptability during this time. RP models were selected for comparison if they have been used on at least 10 patients and if there had been no changes in target structure or OAR naming convention over the previous 3 years to enable ease of comparison. The 4 models that were suitable were CNS non‐overlap (where PTV does not overlap with OAR), oesophagus, prostate (prostate alone, no pelvic nodes) and rectum. In the prostate subsite, the definitive treatment changed from a 40 fraction to 20 fraction approach following published evidence of non‐inferiority with hypofractionated RT,^11^, ^12^, ^13^ which resulted in different doses in the MP and RP plans. However, the comparison was found to be acceptable because the treatment volumes were unchanged, objectives for plan approval remained the same percentage of total dose, and the prostate RP model was used for both standard and hypofractionated plans. Approximately 40–60 historical plans and 10 RP plans were used for comparison in each of the four subsites. All the historical CNS, prostate and rectal MPs used for comparison had been treated dynamically. The CNS plans were IMRT plans, and the Prostate and rectal plans were VMAT plans. Historical oesophageal MPs had been treated with 3D conformal radiation therapy (3DCRT). All RP plans were created dynamically, oesophagus and CNS were IMRT plans, and prostate and rectal plans were VMAT plans. We compared the percentage of MP and RP plans that met national guidelines^8^ in each subsite. As we were comparing four different subsites, only one planning objective for target coverage and 2 OARs were considered for each subsite. A dosimetric comparison was made between the MP and RP plans comparing the median dose per cent and dose range for each constraint.

## Results

A total of 24 models were available for use during the period of this study. Between November 2017 and May 2018, 496 patients were simulated. 217 (43.8%) patients were eligible to be planned with one of the models. Of the 279 patients with no available model, 139 (52.5%) were palliative and 114 (43%) were breast. The remaining 26 patients were from a variety of subsites containing small numbers. The first model was the oropharyngeal model. It took 4 months to train and validate this model. Subsequent models took one week or less to create.

### Clinically acceptable plans

RP was successful in creating a clinically acceptable plan in 87.2% of eligible patients.

### Model performance

**Figure 1:**
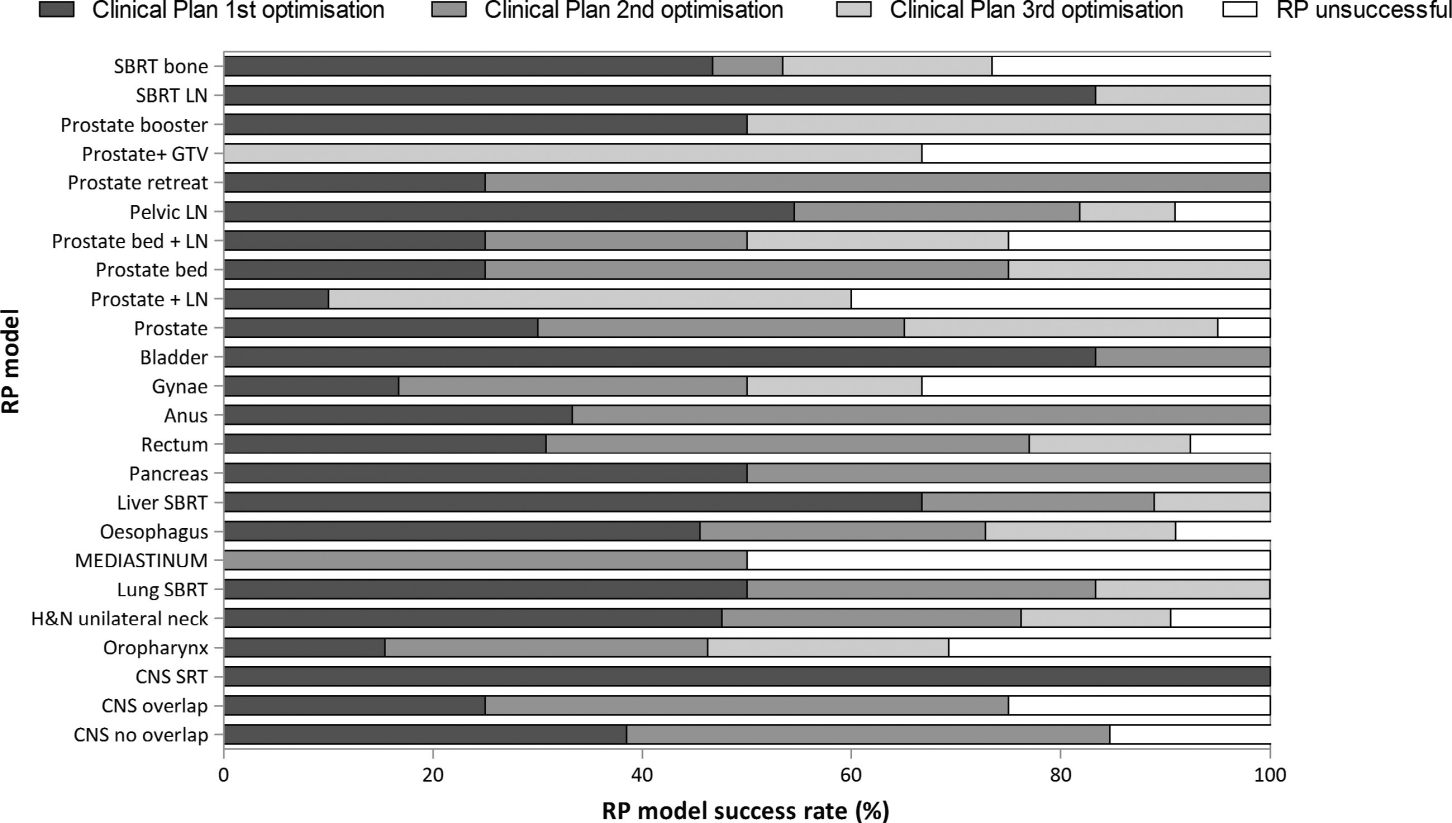
Demonstrating the success rate of RP models and the number of iterations required for each subsite. RP was successful in 87.2% of plans with a clinically acceptable plan being generated in the first iteration in 40% of plans, on the second iteration in 30% (abbreviations: CNS: central nervous system; SRT: stereotactic radiation therapy; H&N: head and neck; SBRT: stereotactic body radiation therapy; Gynae: gynaecology; LN: lymph node; and GTV: gross tumour volume).

The individual success of the models ranged from 50% to 100%, (see Table 1), with 100% success seen in 10 models (41.7%) and more than 90% success in 15 (62.5%) of the models. Only five models (20.8%) had success rates <75%. The percentage of plans per RP model requiring 1, 2 or 3 optimisations is shown in Table 1 and Figure 1. In 40% of plans, success was achieved on the 1st optimisation and in 30% on the 2nd optimisation. RP was deemed unsuccessful (requiring more than 3 optimisations) in 13% of plans. Stereotactic body radiotherapy (SBRT) models had greater success on the first optimisation than the other models. The number of plans used to train each model is recorded in Table 1. There was no correlation between number of plans included in a model and the success of the model.

**Table 1:** Number of patients planned with each RP model, with the percentage success rate of each model and the number of patients (%) requiring 1, 2 or 3 optimisations to achieve a clinically acceptable plan with RP.

	No. of plans used to train model	No of patients on which model was used	RP% success	Clinically acceptable plan on 1st optimisation	Clinically acceptable plan on 2nd optimisation	Clinically acceptable plan on 3rd optimisation
CNS no overlap	68	13	84.6	5 (38.5)	6 (46.2)	0
CNS overlap	42	4	75	1 (25)	2 (50)	0
CNS SRT	68	2	100	2 (100)	0	0
Oropharynx	70	13	69.2	2 (15.4)	4 (30.8)	3 (23.1)
H&N unilateral neck	50	21	90.5	10 (47.6)	6 (28.6)	3 (14.3)
Lung SBRT	36	12	100	6 (50)	4 (33.3)	2 (16.7)
Mediastinum	26	2	50	0	1 (50)	0
Oesophagus	26	11	90.9	5 (45.5)	3 (27.3)	2 (18.2)
Liver SBRT	31	9	100	6 (66.7)	2 (22.2)	1 (11.1)
Pancreas	21	6	100	3 (50)	3 (50)	0
Rectum	32	13	92.3	4 (30.8)	6 (46.2)	2 (15.4)
Anus	24	3	100	1 (33.3)	2 (66.7)	0
Gynae	22	6	66.7	1 (16.7)	2 (33.3)	1 (16.7)
Bladder	27	6	100	5 (83.3)	1 (16.7)	0
Prostate	50	19	94.7	6 (31.6)	7 (36.8)	6 (31.6)
Prostate + LN	30	10	60	1 (10)	0	5 (50)
Prostate bed	32	4	100	1 (25)	2 (50)	1 (25)
Prostate bed + LN	38	8	75	2 (25)	2 (25)	2 (25)
Pelvic LN	21	11	90.9	6 (54.5)	3 (27.3)	1 (9.1)
Prostate retreat	25	4	100	1 (25)	3 (75)	0
Prostate + GTV	50	3	66.7	0	0	2 (66.7)
Prostate Booster	20	2	100	1 (50)	0	1 (50)
SBRT LN	20	6	100	5 (83.3)	0	1 (16.7)
SBRT bone	34	15	73.3	7 (46.7)	1 (6.7)	3 (20)
Total		203	87.2%	81 (39.9%)	60 (29.6%)	36 (17.7%)

CNS, central nervous system; GTV, gross tumour volume; Gynae, gynaecology; H&N, head and neck; LN, lymph nodes; SBRT, stereotactic body radiation therapy; SRT, stereotactic radiation therapy.

### RP and MP Planning efficiency comparison

**Figure 2:**
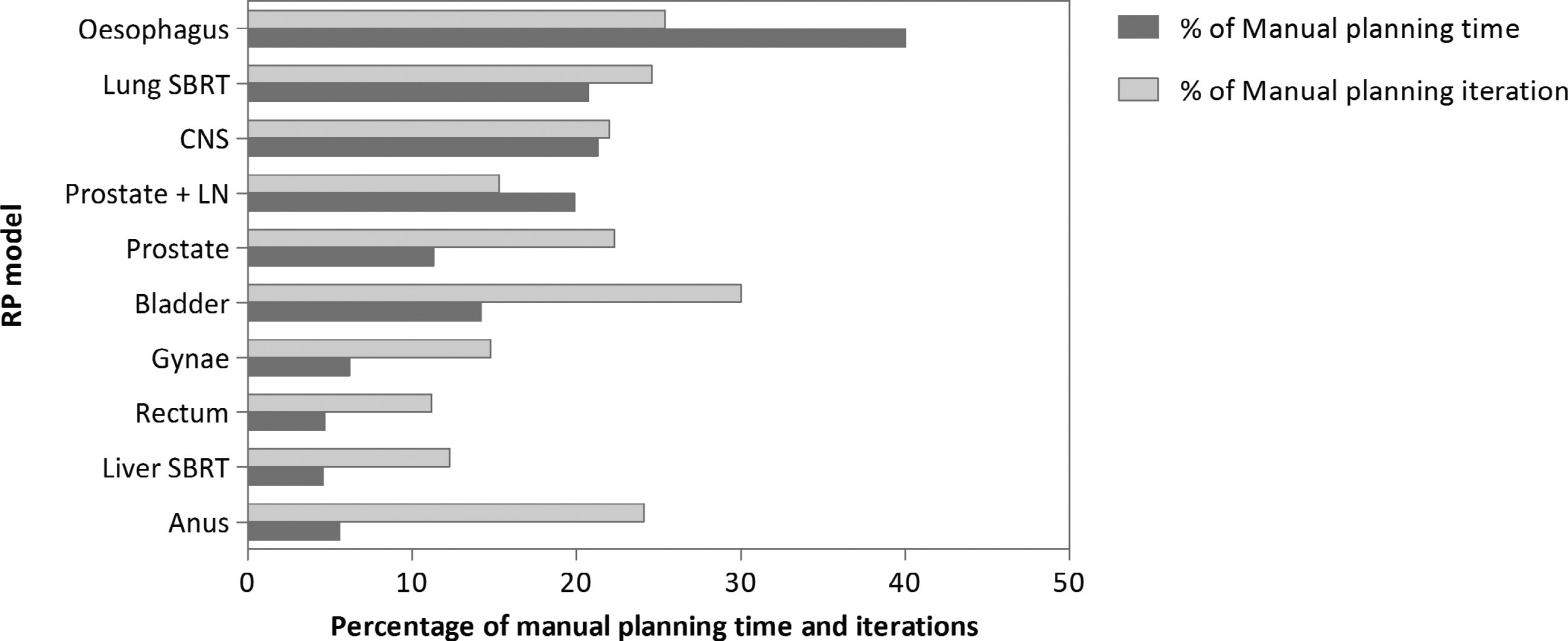
Graph representing the number of iterations and overall planning time with RapidPlan for each subsite as a percentage of number of iteration and overall planning time with manual planning (100%). The average planning time and number of iterations were substantially reduced in all subsites with the use of RapidPlan (abbreviations: CNS: central nervous system; SBRT: stereotactic body radiation therapy; Gynae: gynaecology; and LN: lymph node).

The number of optimisations required to achieve a clinically acceptable plan was reduced by more than 70% with RP compared with MP (see Table 2 and Figure 2). The overall planning time with RP was reduced by up to 95% of MP times with the greatest impact being on rectal, gynaecological and liver SBRT planning. An average rectal plan created with RP took 4.7% of the time taken to create a plan manually. The oesophagus model was the worst performing model in terms of time reduction, but still reduced the overall planning time by 60%.

**Table 2:** Comparison between the average planning time and the average number of optimisations to achieve a clinically acceptable plan for manual planning and RapidPlan for different treatment subsites. The average planning time and number of optimisations were substantially reduced in all subsites with the use of RapidPlan

Model name	Average time manual planning (min)	Average number of optimisations with manual planning	Average time RapidPlan planning (min)	Average number of optimisations with RapidPlan planning
CNS	178	8.3	38.0	1.83
Lung SBRT	185.3	6.5	38.3	1.6
Oesophagus	100	6.3	40	1.6
Liver SBRT	622	12.2	28.75	1.5
Rectum	1137.5	19.3	53.89	2.17
Anus	720	8.3	40	2
Gynae	805	13.5	50	2
Bladder	300	6	42.5	1.8
Prostate	390.7	10.3	44	2.3
Prostate + LN	456.7	15	91	4.5

CNS, central nervous system; Gynae, gynaecology; LN, lymph nodes; SBRT, stereotactic radiation therapy.

### RP and MP Plan Quality comparison

Table 3 demonstrates the achieved target coverage and OAR doses with RP and MP according to recommended guidelines. In all four subsites, the prescribed target coverage was achieved in 100% of RP plans. With the exception of bladder constraints for rectal RP plans, the RP plans were able to achieve dose constraints in 100% of plans.

**Table 3:** National guideline recommended planning objectives for target coverage and 2 OARs per subsite, percentage of MP and RP plans achieving these constraints and median percentage dose and the dose range. RP plans were able to achieve dose constraints for all OARs except for the bladder in the rectal plans.

Treatment site	Prescribed dose	Target/OAR	Planning objectives	% of plans achieving plan objectives		Median % dose to target/OAR (range)	
				MP	RP	MP	RP
Prostate	80Gy/40F	PTV HD	D95% > 98%	100%		100.3% (98.4–101.8%)	
	60Gy/20F	PTV HD	D95% > 98%		100%		100.2% (100–100.5%)
	80Gy/40F	Rectum	V65Gy < 17% V40Gy < 35%	92% 98%		13.27% (3.7–22.8%) 30.2% (21.3–38.9%)	
	60Gy/20F	Rectum	V57Gy < 15% V40.8Gy < 60%		100% 100%		6.1% (2.6–10.2%) 18% (10.5–23%)
	80Gy/40F	Bladder	V60Gy < 30% V40Gy < 50%	98% 100%		11.3% (1.2–30.3%) 22% (2.1–49.2%)	
	60Gy/20F	Bladder	V60Gy < 5% V40.8Gy < 50%		100% 100%		2.7% (0.1–4.8%) 9.5% (2.3–18.1%)
Rectum	50Gy/25F	PTV HD	D95% > 98%	94%	100%	100% (97.4–101.3%)	100.5% (100–101.9%)
		Bladder	V40Gy < 40%	83%	86%	27.4% (12.2–56.8%)	28.3% (3.4–45.4%)
		Small bowel	V45Gy < 195cc	100%	100%	19.8cc (0–96.18cc)	12cc (0.84–92cc)
CNS no overlap	60Gy/30F	PTV HD	D95% > 95%	100%	100%	98.5% (95.9–100.8%)	98.8% (97.2–100.5%)
		Brainstem	Max dose < 54Gy	100%	100%	0%	0%
		Optic chiasm	Max dose < 54Gy	100%	100%	0%	0%
Oesophagus	41.4Gy/23F	PTV	D95% > 95%	93%	100%	98.8% (88.1–103.4%	96.8% (96–100.2%)
		Lung	V20Gy < 20%	96%	71%	8.93% (1.9–24.7%)	15.13% (3–26.1%)
		Heart	V40Gy < 30%	96%	100%	6.15% (2.7–44.2%)	8% (1.6–14.3%)

The grey shade indicates there is no data for this block. All 80Gy/40F prostate plans were planned manually and 60Gy/20F prostate plans were planned with RP.

CNS, central nervous system; MP, manual plan; OAR, organ at risk; RP, RapidPlan.

## Discussion

The ideal radiotherapy treatment plan satisfies both the dose prescription and the normal tissue dose constraints. The process of achieving the optimal dose distribution can be time‐consuming and dependent on the experience and skill of the planner. KBP utilises a database of previous plans to derive a new patient‐specific treatment plan, often with improved target coverage and lower OAR doses. A previous study has demonstrated that less experienced planners are able to achieve plans comparable in quality to an experienced planner when utilising this software.^14^ This approach may significantly reduce planning time with similar or superior plans.^5^, ^6^, ^15^

Initial implementation of RP requires a period of model development, which can be slow and non‐productive. At Royal North Shore Hospital (RNSH), IMRT has been in use since 2001. Since 2006, all treatment subsites have had department‐specific protocols that have been adhered to during the planning process, resulting in low variability in the quality of treatment plans. In an attempt to achieve an ideal treatment plan, historically no limit was set on the number of optimisations, with planners having performed up to 27 optimisations on occasion, resulting in excellent treatment plans but significantly prolonged planning times. As RP models are usually created from a database of IMRT and VMAT plans, palliative patients, usually treated with simple 3D conformal techniques and breast patients, treated with hybrid IMRT and 3DCRT at the time of reporting, did not have models available and made up approximately 50% of the patients treated in the department. This is an area of current research, understanding the potential benefit of utilising more complex planning techniques for breast cancer or palliative radiation which, counter intuitively, may decrease the time in planning if RP models are successfully developed.

In the cohort examined, 43.8% of patients simulated were eligible for RP planning, with an 87% RP success rate. The quality of the RP model depends on the quality of the treatment plans that are incorporated into the model. RP recommends a minimum of 20 plans per model, with additional plans resulting in increased efficacy of the model, with the aim being a single optimisation sign off. There is currently no evidence to suggest the optimal number of plans to be included in a model. In our experience, the effectiveness of the individual models was multifactorial, and not dependent on the number of plans included in the model alone, but also the quality of the plans included in the model and the specificity of the model. The oropharyngeal model (which included all plans with tonsillar and base of tongue cancers) was the first model created with the largest number of plans (70); however, the success rate was only 69%, positioning it as one of our poorer performing models. The gynaecological model, the second model created, was also one of the worst performing models with a 67% success rate. This highlights the learning curve associated with RP implementation, with subsequent models having higher success rates. The gynaecological model included all gynaecological subsites, including intact cervix cancer plans and post‐operative endometrial cancer plans, in one model. The RP recommendation is to include all plans from the same subsite into one model; however, our experience, as demonstrated by the success of the range of genitourinary models which are subdivided based on the treatment protocol, indicates that it may be better to have more specific models. Challenging plans, such as CNS with overlap between PTV and OAR volumes, remain a challenge even with the use of a RP model, confirmed by the lower success rates of these models. SBRT with small treatment volumes and single dose levels are relatively easy to plan and this is reflected in the success of the SBRT RP models.

With all models, the number of optimisations and overall planning times was considerably reduced, with a greater than 80% reduction in planning time in most subsites. Reduction in planning time with the use of RP has been reported in a number of previous studies.^5^, ^6^, ^15^, ^16^ By reducing planning time in a department, more resources are available to create new RP models, refine existing models and complete a greater number of new treatment plans per week. The oesophageal model, which had the smallest decrease in planning time, still reduced planning time by 60%. Oesophageal treatment plans in our department have not routinely been inverse planned, and therefore, only a limited number of inverse plans were available for inclusion in the model library. DVH curves from 3DCRT plans were therefore used to help train the model. RP does not determine the angles of beam entry and therefore when treating the oesophagus, one of the main challenges is keeping the lung V5Gy within acceptable dose constraints.

With KBP, not only is the efficiency of the treatment planning process improved, but the quality of plans may be enhanced, as clinically acceptable PTV coverage is achieved as well as improvements in dose sparing to OARs. Previous studies investigating the use of RP in a variety of subsites, including CNS, head and neck, spine, breast and rectum, all reported comparable or improved target coverage with equivalent or improved OAR doses.^5^, ^7^, ^14^, ^15^, ^17^ In our department, RP reduced the planning time by more than 80% in most subsites, but also produced plans of at least equivalent quality to those manually planned. This combination of efficiency and quality can facilitate more consistent treatment planning within and between institutions. Over time as models are updated and improved, there is potential for further improvement in plan quality and greater department efficiency.

We recognise that there are a number of potential biases in this study. Due to small numbers, only 10 of the 24 subsites were analysed for efficiency and only 4 for quality. As more patients from each subsite are treated, analysis of more subsites would reduce the potential bias. Historical plan data were used for the planning comparison and are therefore not a direct comparison and susceptible to confounders. Change in staff planning skill, improved planning technology and a change in the criteria for plan acceptability must be considered; however, manual plans from the previous 3 years were chosen for comparison to reduce bias as no notable changes in the department technology or criteria for plan acceptability had occurred. To assess plan quality, only 1 planning objective for target coverage and 2 OARs were analysed, which only represents a snapshot of a treatment plan. Median dose percentage was compared instead of individual values, and there were more historical MP plans than RP plans included in the comparison. We acknowledge than the results in this study have several limitations, however it does provide an insight into the early impacts of RP implementation and should be used to guide further research in this area. RP validation should be done in a prospective clinical trial dataset where a direct planning comparison can be done to accurately assess the value of RP as a tool for multicentre clinical trial planning and quality analysis.

## Conclusion

While initially time‐consuming and resource‐intensive to implement, plans optimised with RP demonstrate clinically acceptable plan quality, while significantly improving the efficiency of a department, suggesting RP and its application is a highly effective tool in clinical practice.

## Conflict of Interest

There are no known conflicts of interest associated with this publication.
